# Wafer-Level Filling of MEMS Vapor Cells Based on Chemical Reaction and Evaporation

**DOI:** 10.3390/mi13020217

**Published:** 2022-01-29

**Authors:** Ping Guo, Hongling Meng, Lin Dan, Jianye Zhao

**Affiliations:** 1 Department of Electronics, Peking University, Beijing 100871, China; pingguo@pku.edu.cn (P.G.); l_d@pku.edu.cn (L.D.); 2 Zhongkeqidi Optoelectronic Technology (Guangzhou) Co., Ltd., Guangzhou 510700, China; menghongling@qdgdz.net

**Keywords:** anodic bonding, CSAC, MEMS vapor cells, physics package

## Abstract

Micro-electro-mechanical system (MEMS) vapor cells are key components for sensors such as chip-scale atomic clocks (CSACs) and magnetometers (CSAMs). Many approaches have been proposed to fabricate MEMS vapor cells. In this article, we propose a new method to fabricate wafer-level filling of MEMS vapor cells based on chemical reaction and evaporation. The Cs metals are firstly obtained through the chemical reaction between cesium chloride and barium azide in a reservoir baseplate. Then, the Cs metals are evaporated to the preform through the microchannel plate and condensed on the inner glass surface of the preform. Lastly, the MEMS vapor cells are filled with buffer gas, sealed by anodic bonding, and mechanically diced into three dimensions: 5 mm × 5 mm × 1.2 mm, 4 mm × 4 mm × 1.2 mm, and 3 mm × 3 mm × 1.2 mm. The full width at half maximum (FWHM) linewidth of the coherent population trapping (CPT) signal of the MEMS vapor cells is found to be 4.33 kHz. The intrinsic linewidth is about 1638 Hz. Based on the CPT signal, the frequency stability is 4.41 × 10^−12^@1000 s. The results demonstrate that the presented method of the wafer-level filling of MEMS vapor cells fulfills the requirements of sensors such as CSACs.

## 1. Introduction

Sensors such as chip-scale atomic clocks (CSACs) and magnetometers (CSAMs) are widely used in many fields, such as satellite navigation systems [1,2,3]; global positioning systems (GPS) receivers [4]; precise timing for seismic measurements on the ocean floor related to oil exploration, acoustic sensing, and earthquake detection [5]; and measurement of magnetic fields produced by the heart [6], brain [7], and in space [8]. Generally speaking, CSACs and CSAMs consist of control circuits and physics packages. Traditional physics packages feature a glass-blown vapor cell. However, glass-blown vapor cells have a large spherical shape with a long stem. Even the smallest glass-blown vapor cells reported until now have a diameter of 3 mm (14.1 mm^3^) except for the stem [9]. Besides, the spherical shape makes glass-blown vapor cells difficult to be assembled. With the growing demand for small-volume, low-power-consumption, and high-performance devices, sensors fabricated by micro-electro-mechanical system (MEMS) technologies are becoming more and more popular. As one of the most important components of physics packages, MEMS vapor cells have been pursued by scientists for many years. Compared to traditional glass-blown vapor cells, MEMS vapor cells have the advantages of smaller volume, easier assembly, and higher fabrication efficiency. The first MEMS vapor cell was presented by Liew et al. in 2004; it has comparable performance to a glass-blown vapor cell, with a volume of 4.5 mm^3^ [10].

Many approaches have been proposed to fabricate MEMS vapor cells, which can be categorized into two groups. The first group is about the filling of alkali metals into MEMS vapor cells, such as direct pipetting alkali metals [10,11,12], on-chip chemical reaction of barium azide (BaN_6_) and alkali metal chloride (RbCl or CsCl) [10,13], laser or thermal ablation of encapsulated alkali metals in wax packets [14], on-chip ultraviolet (UV) induced chemical reaction of alkali azide (RbN_3_ or CsN_3_) [15,16], and paste-based dispensers consisting of cesium molybdate (Cs_2_MoO_4_) and Zr-Al alloy powder [17,18]. The direct pipetting alkali metals method requires a high-resolution pipet (nL level). Additionally, pipetting alkali metals into the microcavities of the preform should be conducted in a glove box, which is difficult to be conducted and brings the risk of contaminating the bonding surface of the preform by alkali metals. The on-chip chemical reaction of BaN_6_ and alkali metal chloride produces alkali metal, nitrogen (N_2_), and barium chloride (BaCl_2_) after the sealing of single-chamber MEMS vapor cells [10]. BaCl_2_ appears as white crystals, and it is opaque. BaCl_2_ and the residual chemical precursors remain in the chamber, which obscures the interrogation laser [13]. In order to solve this issue, double-chamber MEMS vapor cells have been presented [13,17,18,19,20,21]. The laser passes through a dedicated interrogation chamber. BaN_6_ and alkali metal chloride are reserved in another chamber called the reservoir chamber. The two chambers are connected by multiple micro-channels. Thus, after the chemical reaction, BaCl_2_ and the residual chemical precursors are kept in the reservoir chamber. Alkali metals are evaporated to the interrogation chamber through the micro-channels. Nevertheless, the extra reservoir chamber makes it difficult to further reduce the volume of the MEMS vapor cell. Most importantly, the barium residue that remains in the MEMS vapor cells recombines with the residual N_2_, decreasing the N_2_ pressure over time and causing a drift in the CPT frequency [22]. Wax packets encapsulating with alkali metals protect the alkali metals from the ambient atmosphere, which is convenient and avoid the risk of contamination during the sealing of MEMS vapor cells. However, encapsulating alkali metals into the wax packets requires extra procedures and direct pipetting alkali metals. In addition, the residual wax in the MEMS vapor cell blocks the interrogation laser. On-chip UV-induced chemical reaction of alkali azide is a good method to dispense alkali metal without the production of other solid matters. However, the alkali azide is hard to decompose even through a long UV exposure [16]. The residue will block out the interrogation laser, too.

The second group is about the sealing technologies of MEMS vapor cells, such as anodic bonding [10,19], Cu-Cu thermocompression bonding [21], and low-temperature indium bonding [23,24]. Anodic bonding, also known as electrostatic bonding or field-assisted bonding, is almost solely applied to the bonding of silicon to Pyrex glass [25]. Cu-Cu thermocompression bonding does not produce any residual gas during the process. By deposition Cu on the bonding surface, the bonding of other combinations of materials is enabled. However, the presence of copper adds thermal magnetic noise in the MEMS vapor cell. Additionally, it is more sensitive to dust contamination than anodic bonding [21]. Based on low-temperature indium bonding, MEMS vapor cells can be fabricated under a temperature as low as 140 °C. However, the process is far more complex, and it uses anodic bonding as well [23,24].

In this article, wafer-level filling of MEMS vapor cells based on chemical reaction and evaporation is proposed. The fabrication and the performance of Cs MEMS vapor cells are detailed. A homogeneous mixture of CsCl and BaN_6_ is obtained through wet mixing rather than dry mixing. Different from the on-chip chemical reaction of the CsCl and BaN_6_ method, the reservoir chambers are integrated on a specially designed structure called a reservoir baseplate. Thus, single-chamber MEMS vapor cells are able to be realized by the proposed method, which makes it possible to achieve smaller MEMS vapor cells. The reaction product BaCl_2_ and the chemical precursors are kept in the chambers of the reservoir baseplate, i.e., obscuring the interrogation laser and frequency drift are avoided after the sealing of the MEMS vapor cells. In addition, the smaller microchannels in the microchannel plate, along with the intimate contact between the preform and microchannel plate, prevent the bonding surface of the preform from contamination during the chemical reaction and Cs evaporation. Through the proposed method, the 216 chambers in the 6-inch wafer are filled with Cs metals successfully at the wafer level at once instead of one by one. The MEMS vapor cells are mechanically diced into three dimensions: 5 mm × 5 mm × 1.2 mm, 4 mm × 4 mm × 1.2 mm, and 3 mm × 3 mm × 1.2 mm. The full width at half maximum (FWHM) linewidth of the coherent population trapping (CPT) signal of MEMS vapor cells is found to be 4.33 kHz. The intrinsic linewidth is about 1638 Hz. Based on the CPT signal, the frequency stability of the prototype CSAC is 4.41 × 10^−12^@1000 s. The results demonstrate that the presented method of the wafer-level filling of MEMS vapor cells fulfills the requirements of sensors such as CSACs.

## 2. Design and Fabrication

**Figure 1:**
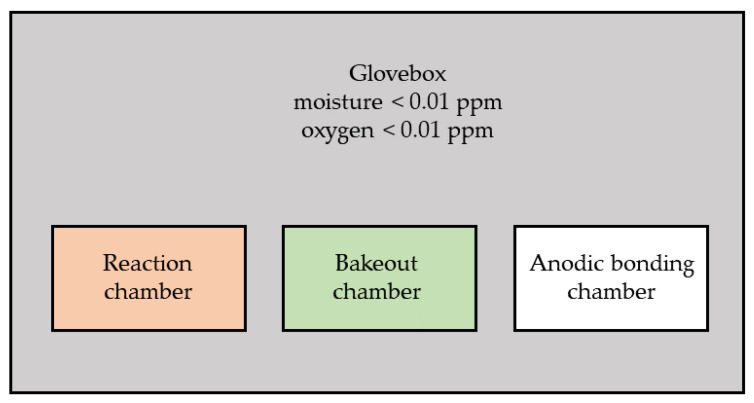
The glovebox integrated with a bakeout chamber, a reaction chmaber, and an anodic chamber.

In this section, the details of the design and fabrication of MEMS vapor cells are linearly introduced. The first anodic bonding process to fabricate the preform is presented in the first subsection. The next subsection presents the production of Cs by chemical reaction of CsCl with BaN_6_ and the evaporation of Cs. Finally, the second anodic bonding of the preform and the top Pyrex glass to the MEMS vapor cell wafer is discussed. Due to the highly reactive properties of Cs metals, a specially designed glovebox is used here, which is integrated with a bakeout chamber, a reaction chamber, and an anodic chamber, as shown in Figure 1. The glovebox is filled with pure N_2_, where oxygen and moisture are kept below 0.01 ppm. The atmosphere in the glove box is more stringent than the one in [10]. The bakeout chamber is used for the desorption of materials such as the Pyrex glass wafer, the silicon wafer, and the preform in vacuum by heating. The reaction chamber is for chemical reaction and Cs metals evaporation. The anodic chamber is for anodic bonding. The integration of the three chambers in a glove box is convenient for the experiment. Almost all of the processes are conducted in the glovebox, except some involving water.

### 2.1. First Anodic Bonding

**Figure 2:**
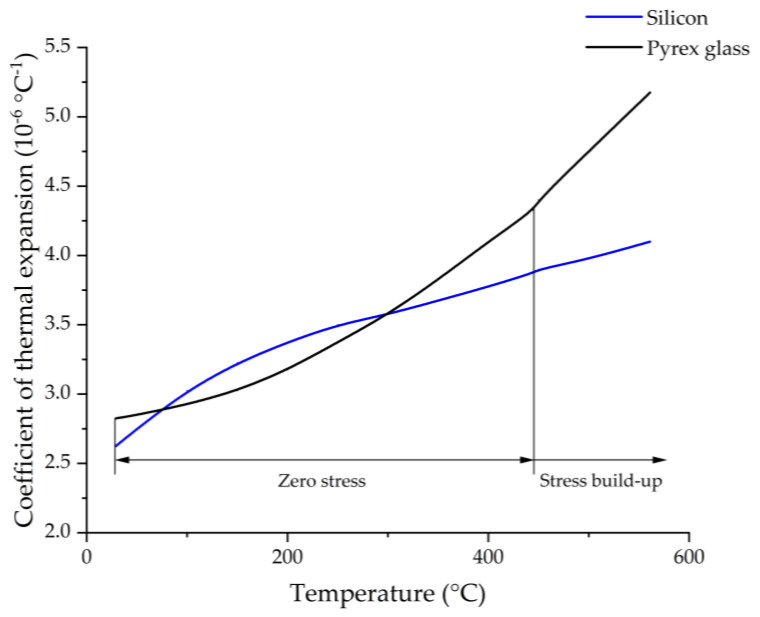
The coefficient of thermal expansion of Pyrex glass and silicon in the range of 20–560 °C.

Anodic bonding is well-established as a joining technology for MEMS devices. Currently, it is also used as the packaging technology for MEMS devices. The materials used to join silicon by anodic bonding should be sufficiently electrically conductive at the bonding temperature. Hence, at the bonding temperature, the material being joined to silicon must contain a significant number of mobile charge carriers [25]. Pyrex glass satisfies the requirements for anodic bonding with silicon and has a good transmission at the wavelength of the Cs D1 line. The coefficient of thermal expansion (CTE) of Pyrex glass and silicon are similar in the range of 20–440 °C, as shown in Figure 2 [26,27]. The CTE mismatch is small, and thus, the stress induced by anodic bonding of silicon to Pyrex glass in the range of 20–440 °C can be kept to a minimum [25,27]. Anodic bonding is generally used to join two pieces together. However, the MEMS vapor cell wafer is a sandwich structure that consists of a bottom Pyrex glass wafer, a silicon wafer with through-holes, and a top Pyrex glass wafer. Thus, the anodic bonding should be adjusted to comply with MEMS vapor cells.

**Figure 3:**
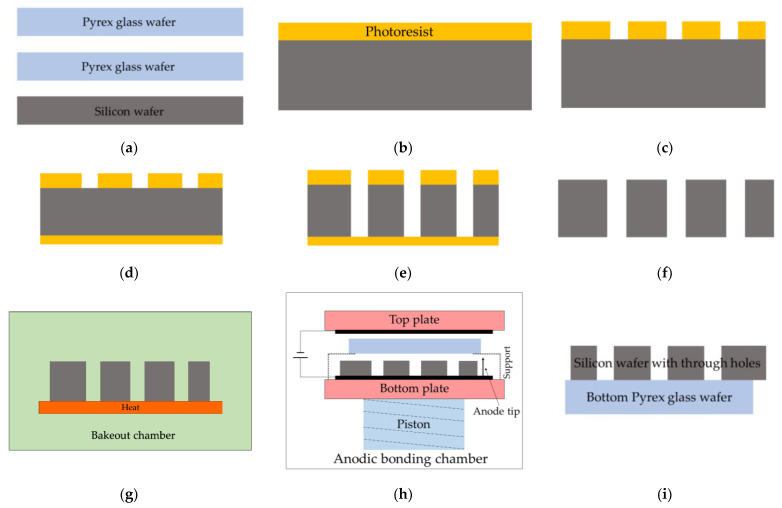
The schematic diagrams of the first anodic bonding. (**a**) Silicon wafer and Pyrex glass wafers preparation; (**b**) Photoresistant coating on the top surface of the silicon wafer; (**c**) Lithograph on the top surface of silicon wafer; (**d**) Photoresistant coating on the bottom surface of silicon wafer; (**e**) Deep reactive-ion etching (DRIE) to fabricate through-holes in the silicon wafer; (**f**) Photoresistant stripping; (**g**) Desorption of the etched silicon wafer and the bottom Pyrex glass wafer; (**h**) The first anodic bonding; (**i**) The preform.

The first anodic bonding of the bottom Pyrex glass to silicon as the preform is shown in Figure 3. Before anodic bonding, through-holes should be etched in the silicon wafer. The flatness, roughness, and cleanness of wafers in anodic bonding are key factors to obtaining good bonding. Root mean square surface roughness of less than 20 nm is eminently suitable [28], although higher roughness values of 50 nm can be tolerated [29]. In order to obtain a better bonding, the root mean square surface roughness, warp, and total thickness variations (TTV) of the double-polished wafers are maintained to be less than 1 nm, 20 μm, and 1 μm, respectively. Contaminant particles will prevent full-scale hermetic sealing. Thus, the wafers should be cleaned by a standard Radio Corporation of America (RCA) clean process [30] and should be used in a clean room. The wafers are prepared under the requirement discussed before, as shown in Figure 3a. The silicon wafer is 6-inch large and 600-μm thick. The glass wafers are 6-inch large and 300-μm thick. The use of thin Pyrex glass for anodic bonding will induce a reduced bow, and thus, the residual stress is decreased [31]. In Figure 3b, a layer of photoresist is spin-coated on the top surface of the silicon wafer. The photoresist is photo-lithographically patterned, as shown in Figure 3c. Another layer of photoresist is spin-coated on the bottom surface of the silicon wafer, as illustrated in Figure 3d. Multiple through-holes with a diameter of 1.5 mm are obtained by deep reactive-ion etching (DRIE) in the 600-μm thick silicon wafer, as presented in Figure 3e. The silicon through-holes are etched by the Bosch process during DRIE, where the deposit phase and the etch phase repeat alternately [32]. The etching rate is about 11 μm/min. The whole DRIE process takes about 1 h to fabricate through-holes in the wafer. Although the etching rate for DRIE varies with the radial distance from the wafer center [33], the etched depth among different holes is the same as the thickness of the silicon wafer. The largest variation of the etched depth among different holes could be estimated by the TTV, which is less than 1 μm. The silicon wafer with through-holes is shown in Figure 3f after stripping the photoresist. It is known that the fluorocarbon passivation layers produced by the deposit phase will react with alkali metals to form alkali fluorides [34,35]. Thus, the fluorocarbon passivation layers should be removed by KOH solutions [19]. After cleaning of the etched silicon wafer, it is transported into the glove box and is outgassed in the bakeout chamber by heating in a vacuum, along with the Pyrex glass wafer, as shown in Figure 3g. The first anodic bonding of the bottom Pyrex glass wafer to the silicon wafer with through-holes is shown in Figure 3h. The silicon wafer with through-holes is put on the bottom plate and is connected to the anode. The bottom Pyrex glass wafer is temporarily supported by a support and is about several millimeters away from the silicon wafer, which ensures better removal of the gas during evacuation. Firstly, the pressure in the anodic bonding chamber is evacuated to be less than 0.001 Pa. Secondly, the support is removed, and the piston pushes up towards the top plate. Hence, the silicon wafer and the bottom Pyrex glass wafer are put in contact. Simultaneously, the bottom Pyrex glass wafer connects to the cathode. Thirdly, the bottom plate and the top plate heat up to 350 °C. At 350 °C, a voltage of 800 V is applied to the anode and cathode. Lastly, the bottom Pyrex glass wafer and the silicon wafer are bonded together to become the preform, as illustrated in Figure 3i. Note that the flats of the silicon wafer and the bottom Pyrex glass wafer should not coincide exactly with each other, or else there will be no room on the silicon wafer for the anode tip during the second anodic bonding. The preform, after the first anodic bonding, cools naturally in the anodic bonding chamber in a vacuum as slow as possible, which will keep the induced thermal stress as small as possible [36]. The preform should be cleaned again to remove the reaction product during the first anodic bonding.

### 2.2. Chemical Reaction of CsCl with BaN_6_ and Cs Evaporation

The chemical reaction of CsCl and BaN_6_ can be described as follows [37]

(1) $$BaN_{6}\overset{200\;{}^{\circ}C}{=}Ba+3N_{2}↑$$

(2) $$Ba+2CsCl\overset{250-300\;{}^{\circ}C}{=}BaCl_{2}+2Cs↑$$

**Figure 4:**
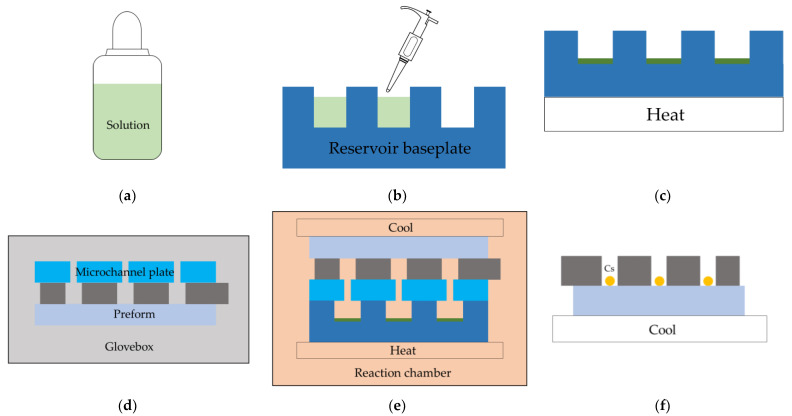
The schematic diagrams of chemical reaction of CsCl with BaN_6_ and Cs evaporation. (**a**) The solution of CsCl and BaN_6_; (**b**) Pipetting the solution to the chambers of reservoir baseplate; (**c**) Evaporation of the solvent; (**d**) Assembly of the preform and the microchannel plate; (**e**) Chemical reaction of CsCl with BaN_6_ and Cs evaporation; (**f**) Holes in the preform filled with Cs metals.

As shown in Equation (1), BaN_6_ decomposes to Ba and N_2_ when it is heated to 200 °C. Additionally, Ba reacts with CsCl at 250–300 °C, as shown in Equation (2). Thus, Cs metals are produced. In order to avoid the double-chamber design of on-chip reaction of CsCl and BaN_6_, a reservoir baseplate with multiple chambers is presented to keep the CsCl and BaN_6_. The chemical reaction and Cs evaporation are shown in Figure 4.

It is difficult to obtain a homogeneous mixture of CsCl and BaN_6_ by dry mixing. However, the solute in the solution is uniformly distributed. As shown in Figure 4a, CsCl and BaN_6_ dissolve in the ionized water as a solution. The solubility in water of BaN_6_ and CsCl at 20 °C is 15.36 g/100 mL [38] and 185.5 g/100 mL [39], respectively. According to Equations (1) and (2), the mass ratio of BaN_6_ and CsCl is 0.657:1. Thus, 1 g BaN_6_ and 2 g CsCl dissolve in 5 mL ionized water, as shown in Figure 4a. In Figure 4b, the solution is pipetted into every chamber of the reservoir baseplate by a 20 μL pipet. A homogeneous mixture of BaN_6_ and CsCl powder is obtained in every chamber through the evaporation of solvent by heating, as illustrated in Figure 4c. The preform and the microchannel plate are first outgassed in the bakeout chamber. Then, they are aligned and pressed into intimate contact in the glove box, as shown in Figure 4d. Next, the chemical reaction and Cs evaporation takes place in the reaction chamber, as shown in Figure 4e. The reservoir baseplate is heated to 300 °C by a heater and the preform is cooled to 0 °C by putting a lid on the preform. The lid is supplied with cold water to cool down the preform. Thus, the reaction product BaCl_2_ and the chemical precursors remain in the chambers of the reservoir baseplate, whereas Cs metals are melted into liquid and evaporated to vapor (the melting point of Cs is 28.5 °C). Cs vapor goes up through the microchannels of the microchannel plate into the holes of the preform. Cs vapor is condensed into droplets on the inner glass surface of the holes in the preform when it contacts the cold surface. The smaller microchannels in the microchannel plate and the intimate contact between the preform and microchannel plate prevent the bonding surface of the preform from contamination during chemical reaction and Cs evaporation. Lastly, the preform filled with Cs metal in every hole is obtained, as shown in Figure 4f.

### 2.3. Second Anodic Bonding

**Figure 5:**
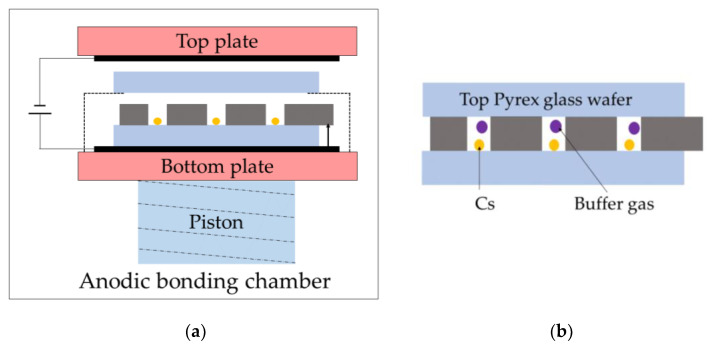
The schematic diagrams of the second anodic bonding. (**a**) The second anodic bonding; (**b**) The MEMS vapor cells wafer filled with Cs and buffer gas.

**Figure 6:**
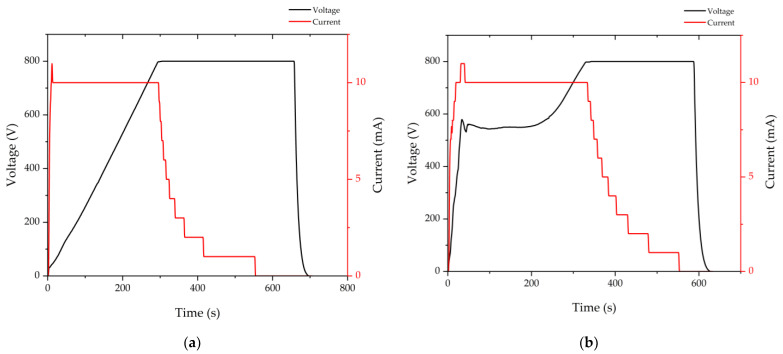
The parameters during anodic bonding. (**a**) The voltage and current during the first anodic bonding; (**b**) The voltage and current during the second anodic bonding.

The second anodic bonding is shown in Figure 5. The second anodic bonding is similar to the first anodic bonding, as shown in Figure 5a. The silicon wafer connects to the anode through a tip. Most importantly, the bottom plate cools the Cs metal down to a solid. The pressure in the anodic bonding chamber is evacuated to be less than 0.001 Pa, and then the chamber is pressurized with buffer gas at a pressure of 100 Torr. The buffer gas is a mixture of Ar and N_2_. The pressure ratio of Ar to N_2_ is 0.795:1. Next, the support is removed, and the piston pushes up toward the top plate. Thus, the top Pyrex glass wafer is attached to the preform, which keeps the holes temporarily hermetic under the piston pressure. Then, the anodic bonding chamber is evacuated again. The temperature is set to be 300 °C. Lastly, the second anodic bonding is completed at a temperature of 300 °C and voltage of 800 V. The MEMS vapor cells’ wafer is shown in Figure 5b. The parameters during anodic bonding are shown in Figure 6. The voltage and current during the first anodic bonding and the second anodic bonding are shown in Figure 6a,b, respectively. During anodic bonding, the voltage is gradually applied to the electrodes to keep the current no more than 10 mA, i.e., if the current is 10 mA, the voltage will keep constant, or else the voltage will increase. As shown in Figure 6, the voltage increases gradually to 800 V and keeps constant at 800 V until the bonding process completes. The current is no more than 10 mA, except for an overshoot in every current curve, which is caused by the rapid increase in current that the program fails to respond to. The magnitude of the current peak strongly depends on the bonding temperature and less on the bonding voltage, sodium concentration of the Pyrex glass, shape of the cathode electrode, and the surface condition [25]. Since the bonding materials are the same during the first and the second anodic bonding, the current behaves similarly. However, the temperature is higher in the first anodic bonding than in the second anodic bonding. Thus, the current increases faster in the first anodic bonding. In order to keep the current no more than 10 mA, the voltage increases slower in the first anodic bonding than in the second anodic bonding, as shown in Figure 6a,b.

## 3. Results and Discussion

### 3.1. The Outlook of MEMS Vapor Cells

**Figure 7:**
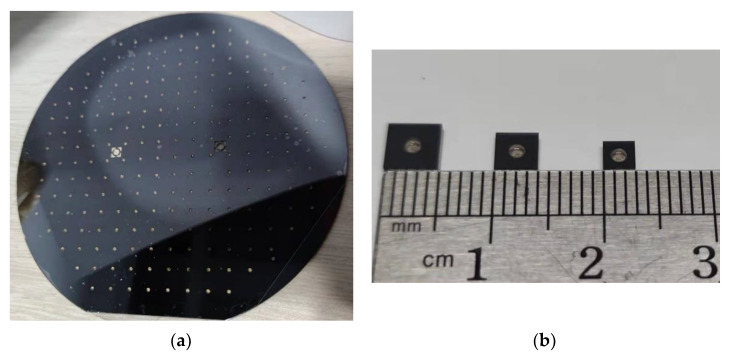
The pictures of the MEMS vapor cells. (**a**) The 6-inch MEMS vapor cells wafer; (**b**) The dimensions of three types of MEMS vapor cells after mechanical dicing.

**Figure 8:**
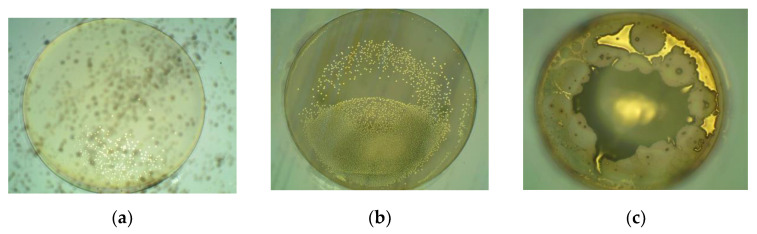
The pictures of the MEMS vapor cells under the microscope. (**a**) An insufficient amount of cesium; (**b**) A reasonable amount of cesium; (**c**) An excessive amount of cesium.

The picture of the 6-inch MEMS vapor cells wafer is shown in Figure 7a. The silicon wafer primary flat is misaligned to the glass wafer primary flat on purpose, which can be seen from Figure 7a. The golden cesium can be seen from the holes in the MEMS vapor cells wafer. The MEMS vapor cells wafer are mechanically diced into separate MEMS vapor cells, as shown in Figure 7b, which only contains one chamber. The dimensions of the three types of MEMS vapor cells are 5 mm × 5 mm × 1.2 mm, 4 mm × 4 mm × 1.2 mm, and 3 mm × 3 mm × 1.2 mm. The pictures of the MEMS vapor cells under the microscope are shown in Figure 8. The golden cesium is filled in the hole of the MEMS vapor cell. There are 216 cells in the 6-inch wafer in total. The cesium is observed in every MEMS vapor cell. However, there are 14 MEMS vapor cells with an insufficient amount of cesium, as shown in Figure 8a. The 14 cells are at the corner of the wafer. A total of 18 MEMS vapor cells have an excessive amount of cesium, as shown in Figure 8c, which locate at the center of the wafer. The other MEMS vapor cells have a reasonable amount of cesium inside, as shown in Figure 8b. The amount of cesium inside the MEMS vapor cells shows a relationship between the location of the wafer. This is probably because the temperature is not uniform in the reservoir baseplate during the chemical reaction, which leads to a different reaction rate among different chambers in the reservoir baseplate. Thus, the amount of cesium accumulated in the MEMS vapor cell differs from each other. This can be improved by a better distribution of temperature. Vicarini et al. reported that more than three-quarters out of 500 MEMS vapor cells from a 6-inch wafer are successfully filled with Cs metals [18]. In the future, the number of MEMS vapor cells fabricated from the 6-inch wafer will increase by reducing the dimensions of cells and the distance between cells.

### 3.2. SEM Images of MEMS Vapor Cells

**Figure 9:**
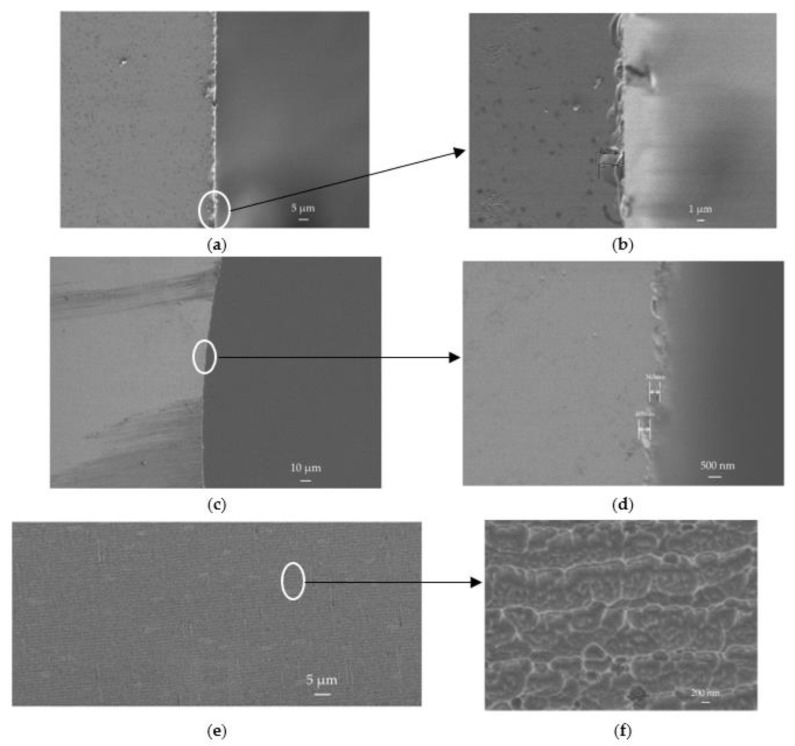
SEM images of MEMS vapor cells. (**a**) The chipping of MEMS vapor cell edge caused by mechanical dicing; (**b**) Magnification of the chipping; (**c**) The edge of the hole etched by Bosch process; (**d**) Magnification of the etched edge; (**e**) The sidewalls of the hole etched by Bosch process; (**f**) Magnification of the etched sidewalls.

Scanning electron microscope (SEM) images of MEMS vapor cells are shown in Figure 9. Figure 9a shows the chipping of the MEMS vapor cell edge after mechanical dicing. The chipping size is about 3.57 μm, as shown in Figure 9b. Additionally, the debris caused by dicing is visible near the MEMS vapor cell edge. The edge of the hole etched by the Bosch process is shown in Figure 9c. The chipping of the etched edge is several hundred nanometers, as shown in Figure 9d. Compared to the edge diced by mechanical dicing, the edge etched by DRIE is smoother. The sidewalls of the hole etched by Bosch process is shown in Figure 9e. The pattern repeats periodically along the etching direction. A more sophisticated image of the repeated pattern is shown in Figure 9f through the magnification of a spot in Figure 9e. The periodic occurrence of the pattern is caused by the Bosch process, where the deposit phase and the etch phase repeat alternately. According to [19], the magnified SEM image in Figure 9f shows that the sidewalls are polished and the fluorocarbon passivation layers are totally removed.

### 3.3. Absorption Spectroscopy of MEMS Vapor Cells

**Figure 10:**
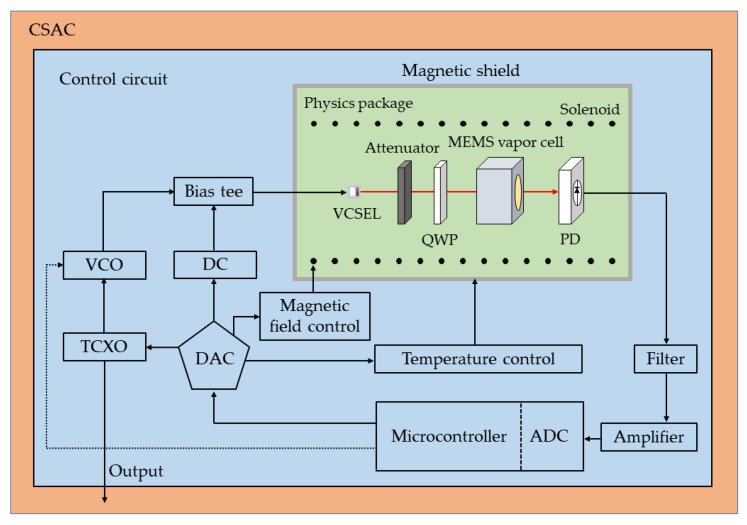
The schematic diagram of the CSAC system.

The performance of the proposed MEMS vapor cell is evaluated by the optimized CSAC system in [40], as shown in Figure 10. The CSAC system consists of two parts: the physics package and the control circuits. In the physics package, the output of the 894 nm vertical-cavity surface-emitting laser (VCSEL) is linearly polarized. The linearly polarized laser passes through the attenuator and the 894 nm quarter-wave plate (QWP). The light intensity is reduced to about 15 μW, and the laser turns into circular polarization. The circularly polarized laser passes through the MEMS vapor cell and interacts with Cs atoms. A photodiode (PD) is used to detect the intensity of the laser passing through the MEMS vapor cell. The solenoid provides a stable magnetic field of about 10 μT to cause hyperfine splitting of Cs atoms. The neighboring CPT resonance is separated by 35 kHz because the neighboring Zeeman sublevels shift by 3.5 kHz/μT for Cs [41]. The physics package is shielded by a magnetic shield to avoid the effect of the geomagnetic field. The operation temperature of the physics package is set to be 80 °C. In the control circuits, a voltage-controlled oscillator (VCO) provides a 4.6 GHz microwave, which is mixed into DC injection current through the bias tee. Driven by the modulated current from the bias tee, the VCSEL outputs a multi-chromatic laser. Its ±1 sidebands are used to probe the CPT signal, whose frequency splitting is 9.2 GHz. The derivative of the CPT signal is obtained by a microcontroller, which processes the data detected by the PD.

**Figure 11:**
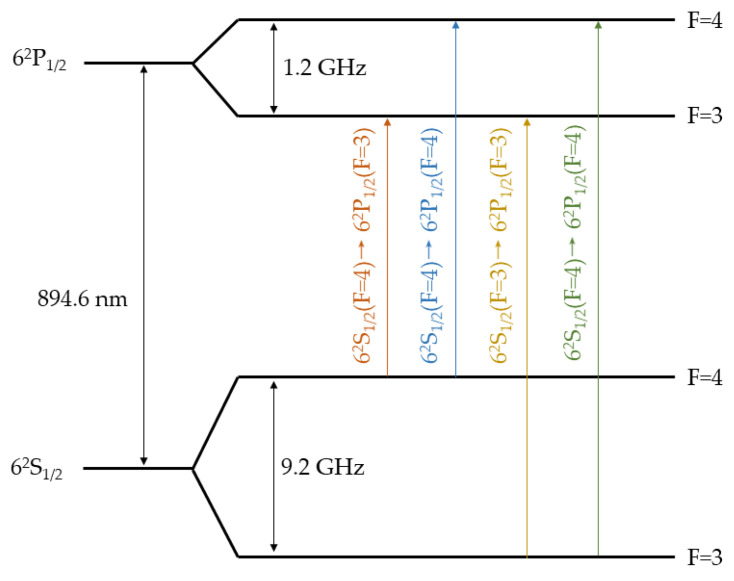
Cs D1 line transition hyperfine structure.

**Figure 12:**
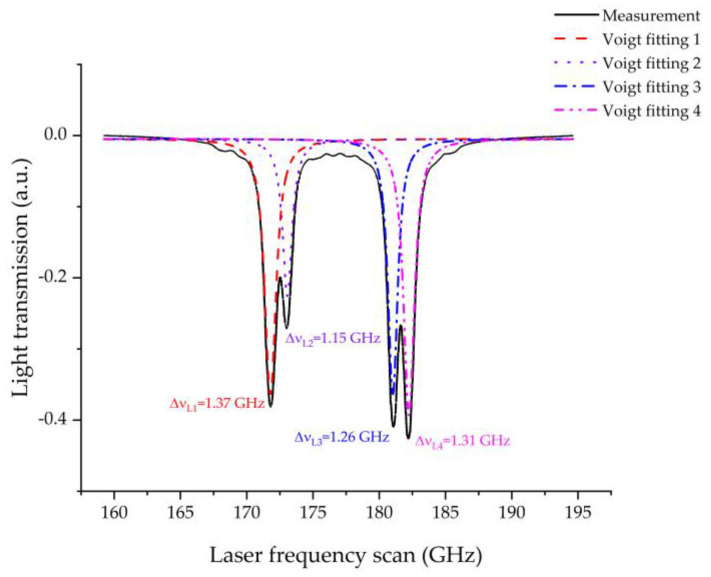
The absorption spectroscopy of the MEMS vapor cell.

The Cs D1 line transition hyperfine structure is shown in Figure 11 [41]. When the output frequency of the VCSEL satisfies the transition requirements, the laser is absorbed partially by Cs atoms, and thus, the light intensity detected by the PD drops. By scanning the output frequency of the VCSEL, the absorption spectroscopy of the MEMS vapor cell is shown in Figure 12. The black line is the measured data, which has four absorption peaks. The four absorption peaks from left to right correspond to the Cs transition 6^2^S_1/2_ (F = 4) → 6^2^P_1/2_ (F = 3), 6^2^S_1/2_ (F = 4) → 6^2^P_1/2_ (F = 4), 6^2^S_1/2_ (F = 3) → 6^2^P_1/2_ (F = 3), and 6^2^S_1/2_ (F = 3) → 6^2^P_1/2_ (F = 4), respectively, which can be found in Figure 11. The red line is Voigt fitting 1 of the Cs transition 6^2^S_1/2_ (F = 4) → 6^2^P_1/2_ (F = 3). The violet line is Voigt fitting 2 of the Cs transition 6^2^S_1/2_ (F = 4) → 6^2^P_1/2_ (F = 4). The blue line is Voigt fitting 3 of the Cs transition 6^2^S_1/2_ (F = 3) → 6^2^P_1/2_ (F = 3). The magenta line is Voigt fitting 4 of the Cs transition 6^2^S_1/2_ (F = 3) → 6^2^P_1/2_ (F = 4). The full width at the half-maximum (FWHM) linewidth of Voigt fitting 1 (Δ*ν_L_*_1_), Voigt fitting 2 (Δ*ν_L_*_2_), Voigt fitting 3 (Δ*ν_L_*_3_), and Voigt fitting 4 (Δ*ν_L_*_4_) is 1.37 GHz, 1.15 GHz, 1.26 GHz, and 1.31 GHz, respectively. Each of the four hyperfine lines have been assumed to share the same Lorentzian linewidth [42]. Thus, the Lorentzian linewidth (Δ*ν_L_*) of the absorption line can be calculated by the mean value of Δ*ν_L_*_1_, Δ*ν_L_*_2_, Δ*ν_L_*_3_, and Δ*ν_L_*_4_, which is 1.27 GHz. The buffer gas pressure in the MEMS vapor cell can be estimated from the Lorentzian linewidth of the absorption line. The D1 line broadening coefficients of Ar (*Γ*_Ar_) and N_2_ (*Γ*_N2_) for Cs are 20.4 MHz/Torr and 18.6 MHz/Torr, respectively [43]. The natural linewidth (*γ*_N_) of Cs is 4.57 MHz [41]. The relationship between Δ*ν_L_* and the buffer gas pressure (*P*) is illustrated by Equation (3) [42].

(3) $$\Delta \nu_{L}=\Gamma_{\mathrm{Ar}}P_{\mathrm{Ar}}+\Gamma_{N_{2}}P_{N_{2}}+\gamma_{N}$$

(4) $$P=P_{\mathrm{Ar}}+P_{N_{2}}$$

where *P*_Ar_ and *P*_N2_ are the pressure of Ar and N_2_ in the MEMS vapor cell, respectively. The ratio of the pressure of Ar to N_2_ is 0.795:1. Substituting these values into Equations (3) and (4), the actual pressure of the buffer gas in the MEMS vapor cell is calculated to be about 65.37 Torr. The difference between the calculated buffer gas pressure and the setting value in the MEMS vapor cell is mainly caused by the measurement error, the instant elevation of the piston right after the gauge reaches 100 Torr, and the effect of the pump. The relationship can be found between the setting pressure value and the actual pressure value. We will try to find an imperial value to let the actual pressure equal the desired pressure of 100 Torr. Moreover, the piston will push up after the buffer gas pressure reaches equilibrium in the anodic bonding chamber.

### 3.4. Frequency Performance of MEMS Vapor Cells

The frequency performance of the MEMS vapor cells is mainly evaluated from two aspects: the CPT signal and the Allan deviation.

**Figure 13:**
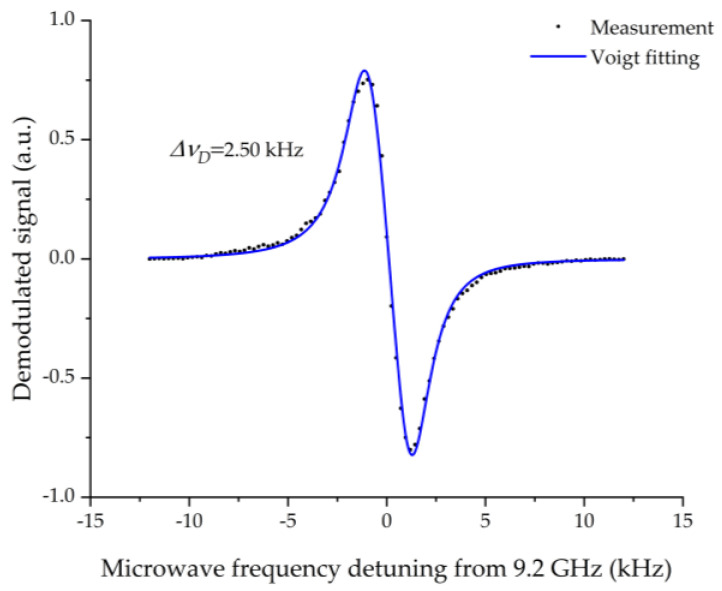
The derivative of the CPT signal detected from the MESM vapor cell.

Figure 13 shows the derivative of the CPT signal detected from the MEMS vapor cell with a light intensity of 15 μW. The CPT resonance is obtained by detuning the modulation microwave frequency from 4.6 GHz, which is half of the hyperfine frequency splitting of the ground-state of the Cs D1 line transition. The black dots are the signal measured from the MEMS vapor cell. The blue line is the Voigt fitting of the measured data. The abscissa distance (Δ*ν*_D_) of the two extrema of the Voigt fitting is about 2.50 kHz, estimated from Figure 13. The FWHM linewidth (Δ*ν*) of the CPT signal can be calculated from the derivative of the CPT signal. In general, the CPT signal is described by the Lorentz curve [44]. The Lorentz function is expressed as follows:

(5) $$f_{L}(x)=\frac{A}{\pi }(\frac{\frac{\Gamma }{2}}{{\left(x-x_{0}\right)}^{2}+{\left(\frac{\Gamma }{2}\right)}^{2}})$$

where *A* is the amplitude, *x*_0_ is the peak center, and *Γ* is the FWHM linewidth of the Lorentz function. The derivative function of Equation (5) is expressed as follows

(6) $$f_{D}(x)=-\frac{A}{\pi }\frac{\Gamma (x-x_{0})}{{\left[{\left(x-x_{0}\right)}^{2}+{\left(\frac{\Gamma }{2}\right)}^{2}\right]}^{2}}$$

**Figure 14:**
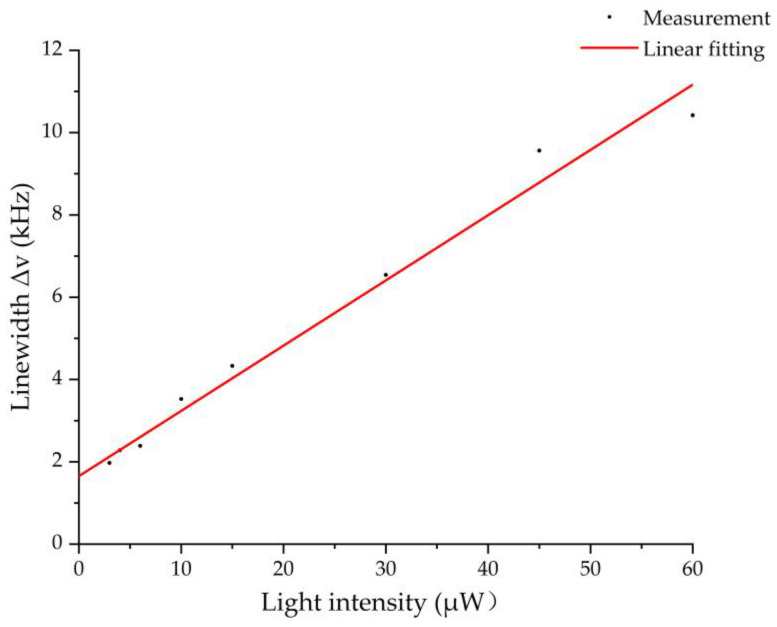
The FWHM linewidth of the CPT signal vs. interrogation light intensity.

The abscissas of the two extrema of the derivative function (6) are $\left(x_{0}+\frac{\sqrt{3}}{6}\Gamma \right)$ and $\left(x_{0}-\frac{\sqrt{3}}{6}\Gamma \right)$. Thus, the abscissa distance of the two extrema of the derivative function is $\frac{\sqrt{3}}{3}\Gamma$, which is $\frac{\sqrt{3}}{3}$ times of the FWHM linewidth of the CPT signal of *Γ*. $\frac{\sqrt{3}}{3}\Gamma$ is estimated to be 2.50 kHz from Voigt fitting. Hence, the FWHM linewidth of the CPT signal of the MEMS vapor cell is calculated to be about 4.33 kHz. The FWHM linewidth can be expressed by [45]

(7) $$\Delta v=\frac{1}{\pi }(\gamma_{2}+\frac{\omega_{R}^{2}}{\Gamma_{e}})$$

where *γ*_2_ is the ground-state coherent relaxation rate, *Γ_e_* is the excited-state coherent relaxation rate of atoms, which is caused by spontaneous decay of excited state and collision gas, and *ω_R_* is the optical Rabi frequency. The dark resonance linewidth depends on the relaxation rate *γ_2_* and a term proportional to the light intensity [46]. The relationship between the FWHM linewidth of CPT signal and interrogation light intensity is shown in Figure 14. The black dots are the measured data by varying the interrogation light intensity. The red line is the linear fitting of the measured data. The intrinsic linewidth (the first term in Equation (7)) of the MEMS vapor cell is about 1638 Hz by extrapolating the light intensity to 0 μW to exclude the light broadening contribution. The intrinsic linewidth of the MEMS vapor cell is larger than others, such as 500 Hz in [47] and 777.4 Hz in [48]. The reason for this is that the MEMS vapor cell has a smaller dimension, lower buffer gas pressure, and higher temperature [48].

**Figure 15:**
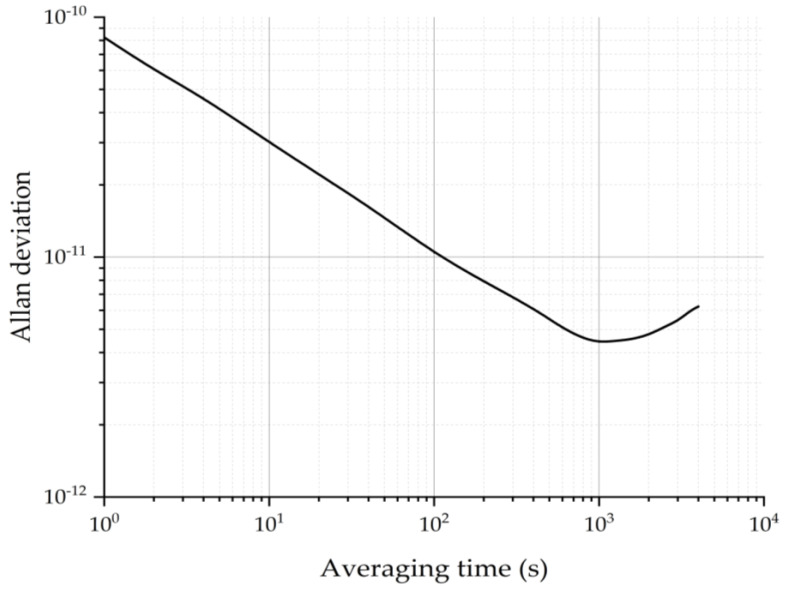
The frequency performance of the MEMS vapor cell.

Based on the achieved derivative of the CPT signal, the frequency performance of the MEMS vapor cell is evaluated by Allan deviation. Allan deviation (σ_x_(*τ*)) is widely used to measure the frequency stability in clocks, oscillators, and amplifiers, which is expressed as follows:

(8) $$\sigma_{x}(\tau )=\sqrt{\frac{1}{2}\langle {\left({\bar{x}}_{n+1}-{\bar{x}}_{n}\right)}^{2}\rangle }$$

where *τ* is the measurement time, $\bar{x_{n}}$ is the n-th fractional frequency average over the measurement time *τ*, and <…> denotes the expectation operator. The frequency of the temperature compensated crystal oscillator (TCXO) is locked to the CPT signal through the derivative. Additionally, the frequency stability of the TCXO is evaluated by a phase noise analyzer, which records the frequency of the TCXO one time per second. The total measurement lasts about 10 h. The measured frequency stability of the prototype CSAC incorporating the MEMS vapor cell is about 8.23 × 10^−11^@1 s and 4.41 × 10^−12^@1000 s, as shown in Figure 15.

In comparison with our previous work, the frequency stability of the CSAC using a traditional glass-blown vapor cell is 4.2 × 10^−12^@1000 s [40]. Compared to the work of other researchers, J. Park et al. presented a CSAC with a MEMS vapor cell having the frequency of 5.2 × 10^−12^@1000 s [49]. The MEMS vapor cells fabricated by the proposed method have a similar performance with other counterparts, and thus, they are a great substitute for the traditional glass-blown vapor cells due to their advantages of smaller volume, easier assembly, and high production efficiency. From the above description, the proposed method is superior to other methods for MEMS vapor cells’ fabrication in the following aspects: (1) The MEMS vapor cells are able to be made smaller due to the single-chamber structure. (2) It is safer to obtain a more homogeneous mixture of CsCl and BaN_6_ through wet mixing. (3) It is more efficient to fill Cs metals into multiple holes in the preform at wafer-level at the same time. (4) The bonding surface is protected from contaminants during chemical reactions and Cs dispensing. (5) The MEMS vapor cell can be filled with arbitrary buffer gas, and there are no chemical precursors or reaction production. Nevertheless, the proposed method requires modifications to the general anodic bonding machine to support the bonding of the sandwich structure. Additionally, keeping the quantity of Cs metals the same among different MEMS vapor cells requires a more uniform temperature distribution in the reaction baseplate.

## 4. Conclusions

An approach for the wafer-level filling of the MEMS vapor cells based on chemical reaction and Cs evaporation is proposed. Additionally, the fabrication process is detailed. The homogeneous mixture of BaN_6_ and CsCl is through wet mixing. Different from other MEMS vapor cells fabrication process, the Cs metals are filled into multiple holes in the preform at the wafer-level at the same time. The process is concise and is convenient to perform. Additionally, the process avoids the risk of contamination of the bonding surface. Although the Cs metal is produced by chemical reaction, the MEMS vapor cell contains only one chamber. Furthermore, no extra reaction product or chemical precursors remain in the holes except for Cs metals. Moreover, the MEMS vapor cells can be filled with arbitrary buffer gass. The single-chamber structure is the basis to make the MEMS vapor cells even smaller. Most importantly, the performance of the fabricated MEMS vapor cells is evaluated through microscope, linewidth, and Allan deviation. The results show that the MEMS vapor cells have a CPT FWHM of about 4.33 kHz and an intrinsic linewidth of about 1638 Hz. The frequency stability is about 4.41 × 10^−12^@1000 s. The results demonstrate high performance of the proposed method for MEMS vapor cells.

## Data Availability

Data sharing not applicable.
